# Environment-induced overheating phenomena in Au-nanowire based Josephson junctions

**DOI:** 10.1038/s41598-021-94720-5

**Published:** 2021-07-27

**Authors:** O. V. Skryabina, S. V. Bakurskiy, A. G. Shishkin, A. A. Klimenko, K. S. Napolskii, N. V. Klenov, I. I. Soloviev, V. V. Ryazanov, A. A. Golubov, D. Roditchev, M. Yu. Kupriyanov, V. S. Stolyarov

**Affiliations:** 1 Institute of Solid State Physics RAS, Chernogolovka, Russia 142432; 2 Moscow Institute of Physics and Technology, Dolgoprudny, Russia 141700; 3 Skobeltsyn Institute of Nuclear Physics, MSU, Moscow, Russia 119991; 4 Dukhov Research Institute of Automatics (VNIIA), Moscow, Russia 127055; 5 Department of Materials Science, MSU, Moscow, Russia 119991; 6 Institute of Nanotechnology of Microelectronics RAS, Moscow, Russia 119991; 7 Department of Chemistry, MSU, Moscow, Russia 119991; 8 Faculty of Science and Technology, MESA+ Institute of Nanotechnology, 7500 AE, Enschede, The Netherlands; 9 Laboratoire de Physique et d’Etude des Materiaux, LPEM, UMR-8213, ESPCI-Paris, PSL, CNRS, Sorbonne University, 75005 Paris, France

**Keywords:** Electronic properties and materials, Superconducting properties and materials

## Abstract

Unlike conventional planar Josephson junctions, nanowire-based devices have a bridge geometry with a peculiar coupling to environment that can favor non-equilibrium electronic phenomena. Here we measure the influence of the electron bath overheating on critical current of several bridge-like junctions built on a single Au-nanowire. Using the Usadel theory and applying the two-fluid description for the normal and superconducting components of the flowing currents, we reveal and explain the mutual influence of the neighbouring junctions on their characteristics through various processes of the electron gas overheating. Our results provide additional ways to control nanowire-based superconducting devices.

## Introduction

Nanowire (NW) based Josephson junctions have a bridge geometry and exhibit significantly different operation regimes as compared to traditional stacked Josephson junctions^1,2^. The small volume of NW and their weak thermal coupling to substrate favors strong out-of-equilibrium phenomena to come in play^3,4^. This offers an interesting opportunity for studying non-equilibrium states in Josephson junctions, and their nontrivial manifestations in the dynamic characteristics.

**Figure 1:**
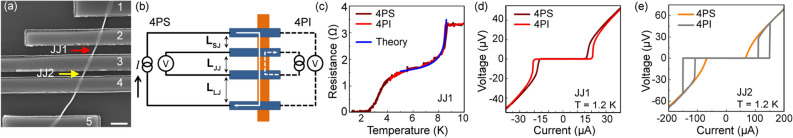
(**a**) SEM image of the Nb/Au-NW/Nb hybrid structure. The red arrow marks JJ1 weak link (*L* = 645 nm), the yellow one marks JJ2 weak link (*L* = 360 nm). Numbers 1–5 are the serial numbers of the Nb electrodes. The scale bar is 1 $\upmu$m. (**b**) A sketch of the two measurement schemes for which the following graphs are given: standard 4-probe scheme (4PS) and inverted 4-probe scheme (4PI). (**c**) Experimental (red and brown lines) and theoretical (blue line) *R*(*T*) dependencies for the JJ1. (**d**, **e**) are *I*(*V*) characteristics, for the JJ1 and JJ2, respectively, measured using different schemes.

In this work, Josephson junctions made of individual Au NW connected to Nb electrodes are studied, which have a multi-terminal design^5–8^, Fig. 1a. We focus on the effect of current input through different electrodes on the junction properties. It is commonly accepted that the conventional 4-probe scheme enables avoiding the influence of the voltage drops at the interfaces between the contact materials on transport characteristics^9^. Here we go a step further and compare different types of 4-probe and 2-probe schemes in combination with different ways of inputs of the normal or superconducting currents into the junction. We have found important differences between the critical current values measured within the standard 4-probe setup (4PS) and both the inverted one (4PI), when the current is supplied through inner Nb electrodes and 2-probe (2P) setup. We show that the differences are due to the transition of the side junctions of the device into a resistive state. Our finding offers an interesting way to control the properties of Josephson junctions locally by purely electric means.

## Results and discussion

The Nb/Au-NW/Nb Josephson structures were fabricated by magnetron sputtering of Nb followed by electron-beam lithography, to form the superconducting electrodes (for details, see Ref.^10^). The transport properties were measured inside a liquid helium cryostat equipped with a variable temperature insert (VTI). The samples were glued onto a copper holder and placed in the VTI filled with He-exchange gas. Several samples were studied; they all showed similar characteristics, presented in Table 1.

We focus on the sample presented in Fig. 1a in which a single Au-NW of $d~\approx 90$ nm in diameter is used. The device contains two Josephson junctions in series, JJ1 and JJ2, made of 645 nm and 360 nm long segments of the same NW delimited by Nb-electrodes 2–3 and 3–4, respectively. Two additional electrodes, 1 and 5, are placed at larger distances away from the junctions. Note that since the NW diameter *d* is much smaller than Josephson penetration depth, $d<< \lambda _J$, the junctions are in one-dimensional regime.

The arrangement of Nb electrodes enables three different ways of the current input. The first measurement scheme corresponds to the standard 4PS method in which the outer electrodes (indicated as 1 and 5 in Fig. 1a) are used for current input, and the inner ones (2 and 3 for sample JJ1, 3 and 4 for sample JJ2, see Fig. 1a) to measure the voltage drop across each junction. The second scheme (4PI) uses the same electrodes but with the inverted roles of current and voltage terminals as compared to 4PS scheme. The current is set through the inner electrodes of each junction, whereas voltage drop is measured across the outer Nb terminals (Fig. 1b). The third scheme is the 2-probe one. The same internal electrodes are used to input the current and to measure the voltage drop (electrodes 2 and 3 for JJ1, 3 and 4 for JJ2, see Fig. 1a).

The evolution *R*(*T*) of the junction resistance with temperature of JJ1 junction is shown in Fig. 1c. It is independent on whether the standard or the inverted schemes have been used and exhibits the behavior typical for all studied samples. At $T = 8.6$ K, the *R*(*T*) exhibits a sharp drop due to the transition of Nb electrodes to the superconducting state. In the temperature range 5 K $\lesssim T \lesssim 8.6$ K, the NW is still in the normal state, and superconductivity in Nb electrodes is suppressed in the vicinity of the contacts with the NW. In this temperature range, the total resistance of the structure is the sum of the resistance $R_{NW}$ of the NW segment located between the two Nb electrodes and the resistances of the contact parts, where the superconducting current is converted into the normal one.

The current conversion is responsible for the further monotonic decrease in the resistance with temperature reduction. The resistance of the measured sample consists of two parts $R(T)=R_{NW}+2 R_{EL}$. Here $R_{NW}=4L_{JJ}\rho _{Au}/(\pi d^{2})$ is the resistance of the middle segment of NW, whereas $R_{EL} = 2\sqrt{\rho _{Au}\rho _{B}}/(\pi d^{3/2})$ is the resistance of the region overlapped by electrode^11^. The temperature dependence of the latter term is associated with specific boundary resistance $\rho _{B}$, which is proportional^12–17^ to $(1-T/T_C)^{-\alpha }$. The exact value of the power $\alpha$ is determined by plenty of processes and significantly depends on the materials and considered temperature range. In our model we assume that temperature dependence mostly correspond with Andreev reflection processes in the considered range of temperatures and $\alpha =1/2$. The approximation of the intermediate part of *R*(*T*) dependence enables one to estimate material parameters of the device^11^. The best fit is shown in Fig. 1c by blue line and corresponds to $R_{NW} = 0.44~\Omega$ and Au resistivity $\rho _{Au}~=~0.43~\upmu \Omega$ cm.

**Figure 2:**
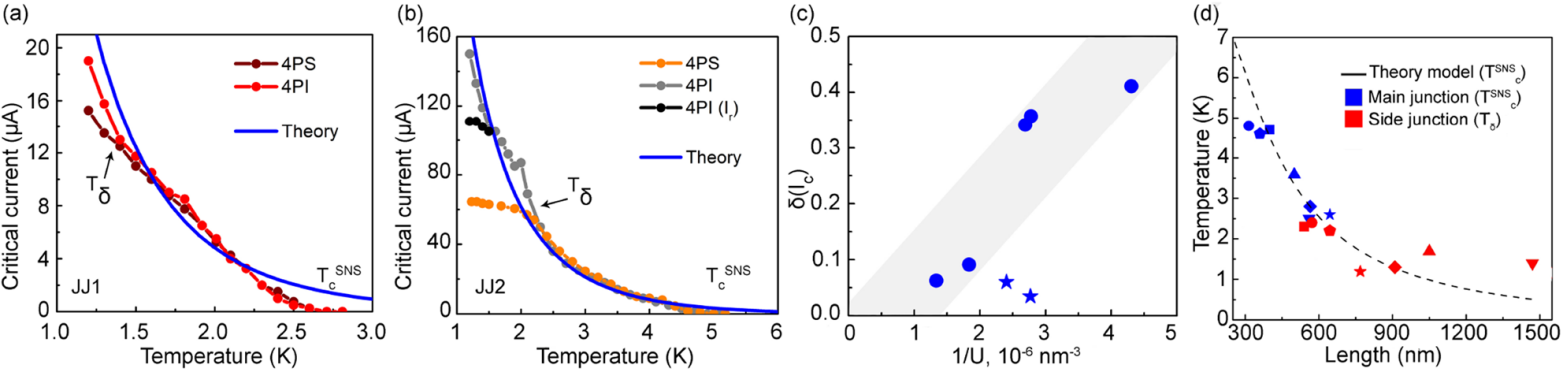
(**a**, **b**) are $I_c(T)$ dependencies of the weak links JJ1 and JJ2, respectively, measured using different schemes. The blue lines are fits by Usadel theory. $T_c^{SNS}$ is the critical temperature of the JJ, $T_{\delta }$ is the temperature below which $I_c(T)$ curves depend on the measurement scheme (see in the text); the temperature dependence of retrapping current is shown with black dots. (**c**) The relative critical current difference $\delta (I_c)$ plotted as a function of the inverse volume of the weak link, 1/*U*. (**d**) Dashed line: expected theoretical dependence of $T_c^{SNS}$ on the JJ length *L*. Blue symbols: experimentally measured $T_c^{SNS}(L)$. Different symbol shapes denote different junctions. Red symbols: measured $T_{\delta }$ of the same junctions presented at positions corresponding to the lengths of their respective side junctions (see explanation in the text).

At $T \lesssim 4$ K, the parts of the NW between Nb electrodes become superconducting by proximity, and further temperature variation of *R*(*T*) is associated with the progressive decrease in the NW part remaining in the normal state. In the junction JJ2, the weak link becomes fully superconducting already at 5 K, whereas in the JJ1 the superconducting state sets in at 2.8 K. Below these temperatures, the junctions show a non-zero supercurrent density that raises with the temperature reduction, Fig. 2a, b.

The current–voltage characteristics *I*(*V*) measured at $T=1.2$ K (see Fig. 1d, e) show all a typical behavior of Josephson junctions; the critical current density $j_c$ = $(1 - 22) \times 10^5$ A/$\hbox {cm}^2$ corresponds well to previously reported values^18^. Remarkably, for both JJ1 and JJ2 junctions, the critical current depends significantly on the chosen measurement scheme. The critical current measured in the inverted 4PI scheme is higher than the one measured using 4PS scheme (JJ1: $I_c^{4PI}~=~20~\upmu$A versus $I_c^{4PS}~=~16~\upmu$A; JJ2: $I_c^{4PI}~=~146~\upmu$A versus $I_c^{4PS}~=~67~\upmu$A). The effect is general and was observed at low enough temperatures in all studied junctions. Furthermore, in the studied temperature range, the current–voltage characteristics of short (< 400 nm) junctions were observed hysteretic, when measured in 4PI and 2P schemes: significantly lower $I_c^{4PI(2P)}$ values were observed on retrapping (decreasing) current. Figure 1e displays this effect for 4PI measurement of JJ2 (grey line). *I*(*V*) measured in 2P scheme are identical to 4PI case and are not shown in figure. The hysteresis is associated with the overheating of the electronic system on retrapping^11^. Though, *I*(*V*) curves measured on the same junction in 4PS scheme (yellow line in Fig. 1e) are characterized by the absence of hysteresis and a relatively low critical current $I_c^{4PS}<I_c^{4PI(2P)}$. The latter is due to the dominant role of the quasiparticles (QPs) injected to JJ2 from already normal, at $I \sim I_c$, side junctions (the role of side junctions is detailed below). The normal QP injection from side junctions holds for both increasing and retrapping branches of *I*(*V*) and explains the absence of hysteresis in 4PS measurements.

**Table 1:** Main parameters of studied devices.

	$L_{JJ} ~(nm)$	*d* (*nm*)	$I_c^{4PS}$ ($\upmu$A)	$I_c^{4PI}$ ($\upmu$A)	$T_c^{SNS} (K)$	$L_{SJ} (nm)$	$L_{LJ} (nm)$	$T_{\delta } (K)$
JJ1	645	90	16	20	2.6	360	760	1.4
A	565	130	8.7	9.5	2.8	910	1120	1.3
B	560	90	10.5	13	2.5	1470	1550	1.4
C	500	120	18.5	23	3.4	1050	1580	1.7
D	400	110	44	93.5	4.7	540	580	2.2
JJ2	360	90	67	146	4.6	645	1600	2.2
E	315	120	67	124	4.8	570	950	2.3

From left to right: Sample names, length $L_{JJ}$ and diameter *d* of NW, critical currents $I_c^{4PS}$ and $I_c^{4PI}$ measured in 4PS and 4PI schemes, critical temperature $T_c^{SNS}$ of the superconducting transition, lengths of the shortest $L_{SJ}$ and longest $L_{LJ}$ side junctions, temperature $T_{\delta }$ (defined in the text).

The results of measurements are summarized in Fig. 2a, b, where the temperature evolution of the critical currents of two junctions are presented for both 4PS and 4PI schemes. Observable differences between critical currents $I_c^{4PS}$ and $I_c^{4PI}$ are revealed below some characteristic temperature $T_{\delta }$ (which is $T_{\delta }=2.2$ K, for the junction JJ2, and $T_{\delta }=1.4$ K, for the JJ1). In general, at $T<T_{\delta }$, the relative difference $\delta (I_c)=\frac{I_c^{4PI}-I_c^{4PS}}{I_c^{4PI}}$ between the values of the critical currents determined by standard and inverted measurement setups is non-zero and depends on the junction lengths and temperature. At $T>T_{\delta }$, the critical current becomes independent of the measurement scheme.

The observed $I_c(T)$ characteristics were examined using the microscopic Usadel approach. The device was represented as a SN-N-NS structure in the bridge geometry with diffusive normal metal of a thickness *d*. The diffusive regime is a reasonable assumption, since the mean free path of electrons is limited by the diameter of NW, leading to a decrease in the effective coherence length (for more details please see SI).

Since the geometrical parameters of the structure are known from SEM images and the resistivity of the gold and critical temperature of Nb-electrodes are determined independently from *R*(*T*) fits, there are only two unknown parameters left: the effective coherence length $\xi$ and the boundary parameter $\gamma _{B}$. Numerical calculations result in reasonable fits of experimental $I_c(T)$ curves, for both junctions (blue lines in Fig. 2a, b). Moreover, both fits are obtained with a single set of material parameters: $\xi = 56$ nm and $\gamma _B=9.4$. Such value of $\gamma _B$ means that the Au/Nb interfaces are low-transparent^19–25^, as could be anticipated for the ex-situ technological process of the preparation of the junction.

According to the estimated coherence length, all studied junctions operate in the so-called long diffusive regime, $L \gg \xi$, for which the relevant parameter is the Thouless energy $E_{Th} = \hbar D/L^2$, where *D* is the diffusion coefficient of the weak link material^26^. The corresponding Thouless temperature $T_{Th}=E_{Th}/k_B$ is $\simeq$ 1.3 K for JJ2, for instance, that is close to $T_\delta \approx 2.2$ K in this junction. The fact that the two temperatures do not match exactly is not surprising. It is a common problem in the experiments on long junctions^27,28^, and is often attributed to non-equilibrium processes.

A good agreement between theory and experimental $I_c(T)$ data obtained in 4PI scheme means that in the inverted geometry the value of the critical current is almost the same as expected for an ideal Josephson junction at equilibrium. Thus, the measurements in 4PS scheme reveal some non-equilibrium effects, which become strong at low enough temperatures. It should be noted, that comparison of $I_c(T)$ dependence for the inverted setup with the theoretical fit reveals an additional effect: in the range of temperatures between 1.5 K and 2.5 K there is a significant hump on the experimental dependence, which distinguishes it from truly exponential curve. This kind of hump on $I_c(T)$ dependence is observed on the other short Nb/Au-NW/Nb junctions too.

The observed non-equilibrium properties can be qualitatively understood considering two kinds of carriers: superconducting condensate and normal QPs. These two components are coupled to each other by Andreev reflection, but due to a finite value of the energy relaxation time their respective chemical potentials may differ^17^. In the frame of this two-fluid model, the difference between the direct and inverted setups is in the way the external currents are coupled to normal and superconducting components of the system. In the 4PS setup, the current is injected along a NW, and one actually injects the normal current in the junction area, while the measurement of $I_c(T)$ reflects the superconducting component, i.e. the difference between chemical potentials of Cooper pairs. In the inverted scheme, on the contrary, a supercurrent is injected into the wire, while the voltage drop due to the QP component is measured. Finally, in the 2-probe mode, one injects a supercurrent and measures the voltage across the superconducting condensate (see Fig. S2 in SI).

The two-fluid concept qualitatively explains the features observed in *I*(*V*) characteristics (Fig. 1d,e). These graphs are the same for both 2P and 4PI methods, with hysteresis of $I_c$ and same critical current values. The reason is the overheating of the superconducting component, while one measures the voltage drop across the normal component. The latter has more efficient heat removal channels as compared to the superconducting one and thus remains in a better thermal equilibrium. In the 4PS scheme, the current is injected to the junction through the long parts of the NW which are in the normal state. This normal current causes a permanent QP overheating in the studied junction. As a result, the critical current in 4PS scheme is significantly lower as compared to 2P or 4PI ones, the *I*(*V*) characteristic is non-hysteretic.

We can now understand better how different parameters affect $\delta (I_c)$. Since $\delta (I_c)$ is related to the overheating phenomena and to the heat exchange, its amplitude and the temperature $T_{\delta }$ at which it appears should depend on the length of the junction, its environment as well as on microscopic mechanisms in play. For instance, the efficiency of the heat exchange via electron-phonon mechanism has strong temperature dependence $\sim T^{(4-5)}$ in the dirty limit^29^ (Supplementary Materials) and provides good thermal equilibrium between electron and phonon baths at temperatures above $\sim$ 1K^1,2,30,31^. Our experimental conditions are close to the case when the exchange between the electron and phonon baths is proportional to $U T^4$, where *U* is the volume of the NW^29^. Other channels of the heat removal from the system are significantly limited, due to a poor link of the NW to substrate and to superconducting electrodes.

Figure 2c shows the evolution of $\delta (I_c)$ measured at $T=1.5$ K as a function of the inverse volume 1/*U* of the intermediate segment of NW. There, the general trend is a rising $\delta (I_c)$ vs 1/*U* dependence, as could be expected. However, it does not hold for thin long junctions (data points presented as stars). This means that in thin long junctions spatial gradients of the overheating effect appear, and the electronic system inside the weak link cannot be considered as homogeneous anymore.

Since the electronic bath of a given junction is coupled to the environment mainly through the NW, the state of the neighbouring side junctions SJ and LJ matters. The effect of the side junctions on the electron bath is summarized in Table 1 and Fig. 2d. Blue symbols denote the critical temperature $T_c^{SNS}$ of several measured junctions. These data points expectedly follow the theoretical $T_c^{SNS}(L)$ curve obtained from Usadel theory using a set of parameters determined above (dashed line). Red symbols mark temperatures $T_{\delta }$ measured in the same junctions. These data points are plotted for each studied JJ at the positions corresponding to the lengths of their respective shortest side junctions. The data points shifted in this way follow a generic $T_c^{SNS}(L)$ dependence, pointing towards a correlation between $T_{\delta }$ measured in JJ and the transition of their side junctions to the superconducting state. Note that for the junction JJ2 (645 nm, star symbol), $T_{\delta }$ was plotted at the position of its longer junction 1–2 (760 nm) and not of its shortest side junction JJ1 (360 nm). The latter is shorter than JJ2, has a higher $T_c^{SNS}$ and $I_c$, and thus cannot be involved in the overheating processes that we discuss now.

The revealed correlation can be interpreted in terms of coupling of JJs with their side junctions. Let us consider a side junction longer than the main one. At high enough temperatures, this side junction is in the normal (resistive) state; it represents a reservoir of normal QPs. The injection of these QPs to the main (superconducting) junction certainly overheats the electronic bath of the latter. However, it is not related to the current injection scheme and thus, the difference between $I_c^{4PS}$ and $I_c^{4PI}$ remains undetectable. When the temperature is lowered, a side junction undergoes a superconducting transition and ceases generating normal QPs. However, its critical current is lower than that of the main junction. In 4PS scheme, the current is injected through this weak side junction. At the critical current of the main junction, the side junction is obviously in the resistive AC-Josephson regime. As a result, there is a voltage drop and overheating reflected in the difference between $I_c^{4PS}$, which is lower, and a higher $I_c^{4PI}$ (as in 4PI the current is injected through superconducting Nb-electrodes). In this “low temperature” regime, a significant (oscillating) contribution to the total current exists. Within this AC-Josephson picture, it becomes immediately clear why side junctions shorter than the main ones (as it is the case of JJ2 for the junction JJ1) do not contribute to the overheating: They have a higher $T_c^{SNS}$ and $I_c$ and remain in the superconducting state.

To summarize, we observed non-equilibrium effects in Josephson junctions containing single Au NWs and studied their origins. In a standard four-probe measurement scheme, the revealed decrease of the critical current was analyzed within the framework of the two-fluid model and identified as due to overheating of the electronic system by injection of a normal QPs into the junction from the current leads. This observation demonstrates that the heat removal from the superconducting part of the electronic system is significantly less efficient that from the normal part. We also found that when several superconducting junctions are put in series, the overheating phenomena in a given junction can be affected by AC-Josephson processes in its neighbouring (side) junctions.

The possibility to control the properties of NW-based Josephson junctions by injecting at will normal or correlated QPs from current-biased side junctions opens a way for experimental studies of non-trivial out-of-equilibrium quantum phenomena. The local character of this all-electric control is also promising for applications of nanowires in electronic circuits^32–34^, where the miniaturization and performance increase are essential challenges. This trend is specifically important for superconducting electronics^35–38^.

## Methods

Gold nanowires were obtained by templated electrodeposition technique. Anodic aluminum oxide (AAO) templates were fabricated by anodization of high-purity (99.99%) aluminum foils at 120 V in 0.3 M oxalic acid at a temperature of 0 $^\circ$C as described elsewhere^39^. Anodization was stopped when a thickness of porous oxide layer reached 50 $\upmu$m. The remaining aluminum was dissolved in an aqueous solution containing 0.5 M $\hbox {CuCl}_2$ and 0.5 M HCl. To obtain oxide film with through-hole pores the barrier oxide layer was removed by chemical etching in a 3 M $\hbox {H}_3$ $\hbox {PO}_4$ aqueous solution at room temperature. Then, a 200 nm thick gold layer was deposited onto the bottom side of the AAO film by RF magnetron sputtering. Electrodeposition was performed at room temperature in a three-electrode electrochemical cell from a commercial electrolyte Ecomet 04-ZG (Russian Federation) containing buffered 50 mM [Au(CN)$_2$]$^-$ (pH 6). During electrodeposition the electrolyte was agitated by a magnetic stirrer. The platinum wire ring with a diameter of 3 cm served as a counter electrode. Electrodeposition was performed in a potentiostatic mode at the deposition potential of − 1.0 V versus saturated (KCl) Ag/AgCl reference electrode. The deposition pulse of − 1.2 V during 0.1 s was applied to stimulate instantaneous gold nucleation. Electrodeposition was stopped when metal started to grow onto the top surface of template. In order to extract the nanowires from the template, AAO was selectively dissolved in 3 M NaOH. For this purpose, Au/AAO nanocomposite was placed for 12 h into the etching solution. Then the solution was changed to freshly prepared one and the sample was stored in it for 12 h again. Finally, the nanowires were washed by decantation 5 times with deionized water. Then the nanowires were washed 5 times with isopropanol, 5 times with acetone, 3 times with heptane and suspended in heptane by ultrasonication^40,41^.

## Supplementary Information

**Supplementary Information 1.**

## Data Availability

The datasets generated during and/or analysed during the current study are available from the corresponding author on reasonable request. Full dataset associated with this article is available in Table 1.
