# Author Correction: Environment-induced overheating phenomena in Au-nanowire based Josephson junctions

**DOI:** 10.1038/s41598-021-96180-3

**Published:** 2021-08-17

**Authors:** O. V. Skryabina, S. V. Bakurskiy, A. G. Shishkin, A. A. Klimenko, K. S. Napolskii, N. V. Klenov, I. I. Soloviev, V. V. Ryazanov, A. A. Golubov, D. Roditchev, M. Yu. Kupriyanov, V. S. Stolyarov

**Affiliations:** 1 Institute of Solid State Physics RAS, Chernogolovka, Russia 142432; 2 Moscow Institute of Physics and Technology, Dolgoprudny, Russia 141700; 3 Skobeltsyn Institute of Nuclear Physics, MSU, Moscow, Russia 119991; 4 Dukhov Research Institute of Automatics (VNIIA), Moscow, Russia 127055; 5 Department of Materials Science, MSU, Moscow, Russia 119991; 6 Institute of Nanotechnology of Microelectronics RAS, Moscow, Russia 119991; 7 Department of Chemistry, MSU, Moscow, Russia 119991; 8 Faculty of Science and Technology, MESA+ Institute of Nanotechnology, 7500 AE, Enschede, The Netherlands; 9 Laboratoire de Physique et d’Etude des Materiaux, LPEM, UMR-8213, ESPCI-Paris, PSL, CNRS, Sorbonne University, 75005 Paris, France

Correction to: *Scientific Reports* 10.1038/s41598-021-94720-5, published online 27 July 2021

The Acknowledgments section in the original version of this Article was incomplete.

"The part of nanowire growth was supported by RFBR 19-02-00981, a theoretical part was supported by RSF 20-69-47013. O.V.S. acknowledges the support of the scholarship of the President of the Russian Federation for the study of electronic transport of the samples. D.R. acknowledges COST Action CA16218—Nanoscale Coherent Hybrid Devices for Superconducting Quantum Technologies."

now reads:

"The part of nanowire growth was supported by RFBR 19-02-00981, a theoretical part was supported by RSF 20-69-47013. O.V.S. acknowledges the support of the scholarship of the President of the Russian Federation for the study of electronic transport of the samples. D.R. acknowledges COST Action CA16218—Nanoscale Coherent Hybrid Devices for Superconducting Quantum Technologies. The work is partially supported by the Twinning project SPINTECH under Grant Agreement Number 810144."

The original Article has been corrected.

