# Supplementary material for: Environment-induced overheating phenomena in Au-nanowire based Josephson junctions
Source: Sci Rep. 2021 Jul 27;11:15274. doi: 10.1038/s41598-021-94720-5 (PMC8316400; doi:10.1038/s41598-021-94720-5)
Supplement: Supplementary file 1 — Supplementary Information 1. [file 41598_2021_94720_MOESM1_ESM.pdf]

Supplementary Information. Environment-induced overheating phenomena in Au-nanowire based Josephson junctions

O.V. Skryabina, S.V. Bakurskiy, A.G. Shishkin, A.A. Klimenko, K.S. Napolskii, N.V. Klenov, I.I. Soloviev, V.V. Ryazanov, A.A. Golubov, D. Roditchev, M.Yu. Kupriyanov, V.S. Stolyarov

Electrochemical preparation and characterization of Au nanowires

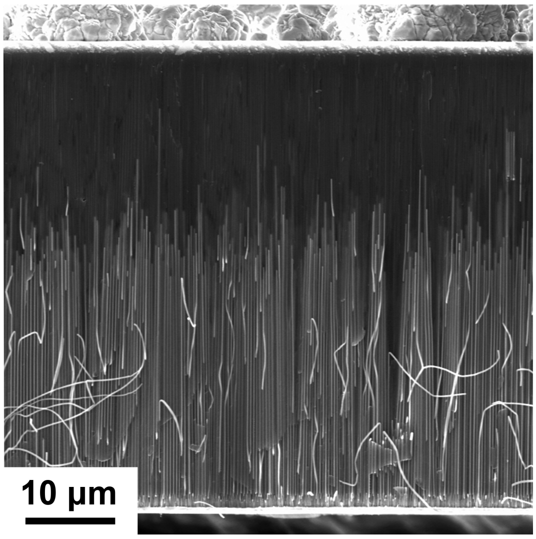

Figure S 1: A cross-sectional SEM image of Au/AAO nanocomposite.

Electron transport measurements

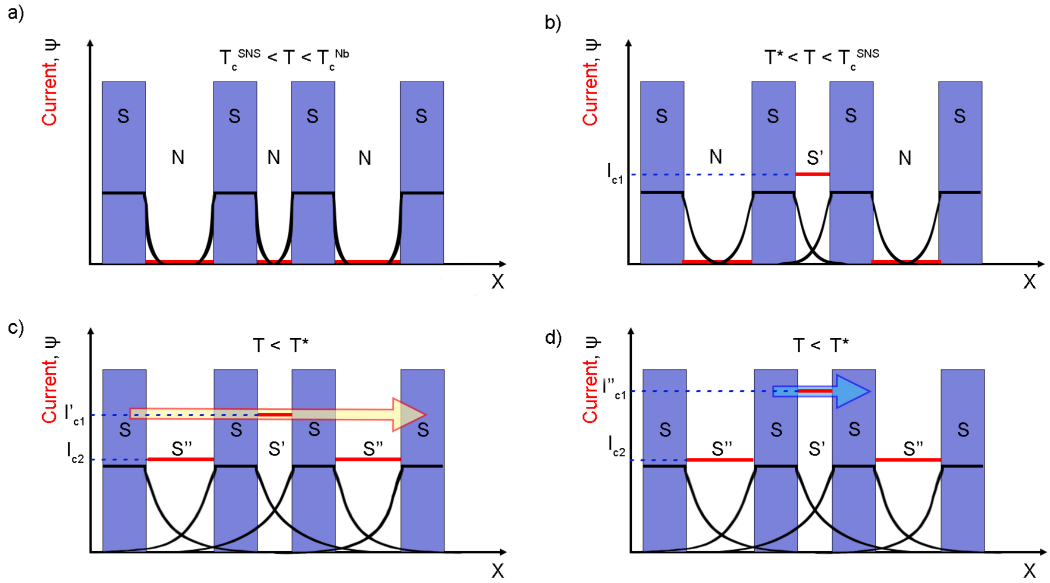

Figure S 2: Schematic diagram of the variation of the wave function amplitude (black curves) and the critical current (red lines) in the SNS Josephson junction based on a single nanowire. The considered weak link is in the center of the structure. The abscissa is the coordinate along the long axis of the nanowire. (a) $T_c^{SNS} < T < T_{Nb}$, the superconducting current is zero. (b) $T^* < T < T_c^{SNS}$, a superconducting current flows through the weak coupling, the side electrodes are in a normal state. (c) $T < T^*$, 4-probe measurement scheme. (d) $T < T^*$, inverted 4-probe or 2-probe measurement schemes.

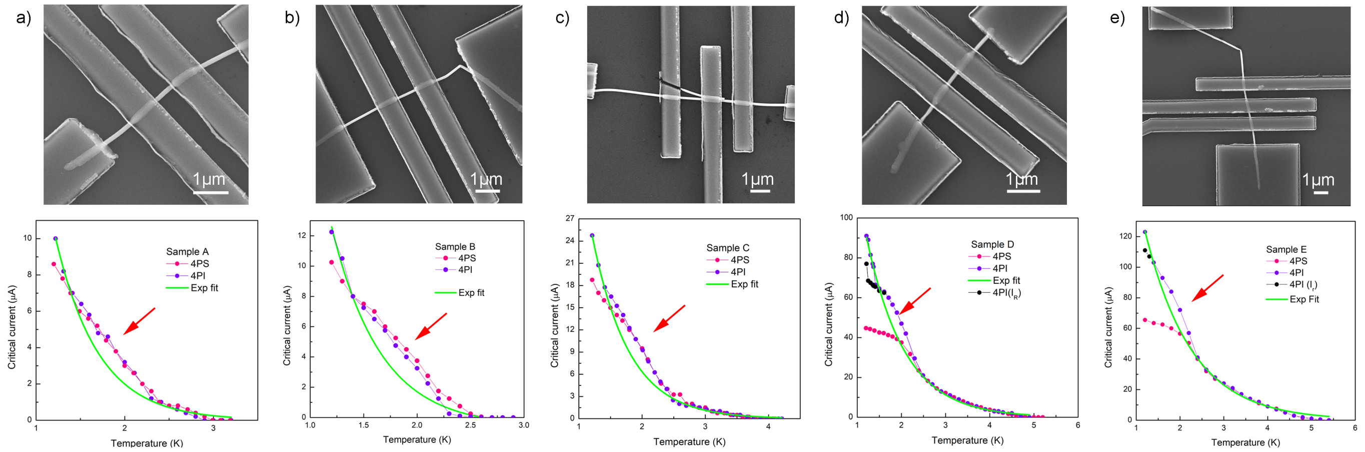

Figure S 3: All data of SEM images and $I_c(T)$ characteristics for samples A-E from the Table 1. Pink curves are for the 4PS case, and violet are for the 4PI one. Black dots correspond to the retrapping current. Green line is the exponential fit of the dependence excluding the mentioned interval of temperature. The red arrows show the hump of $I_c(T)$ which appears in the range of temperatures (1.5 K-2.5 K).

Usadel approach

We consider the problem in the frame of Usadel equations for normal metal wire

$$\frac{\xi^2}{\omega G_\omega}\frac{d}{dx}\left(G_\omega^2\frac{d}{dx}\Phi_\omega\right)-\Phi_\omega=0,\ G_\omega=\frac{\omega}{\sqrt{\omega^2+\Phi_\omega\Phi_\omega^*}}. \tag{1}$$

where $\omega=(2n+1)T/T_c$ are normalized on πT_c Matsubara frequencies, and ξ is coherence length in normal metal. We consider nanowire as normal layer with thickness d and solve equation Eq. 1 in the segment between electrodes.

In the contact regions, the proximity effect is taken into account by considering the pair amplitude:

$$\Phi_\omega(\pm\infty)=\frac{G_s}{(G_s+\gamma_{BM}\omega)}\Delta e^{\pm i\varphi/2}, \tag{2}$$

where

$$G_s=\frac{\omega}{\sqrt{\omega^2+\Delta^2}};\quad \gamma_{BM}=\gamma_B d/\xi,$$

$\omega=(2n+1)T/T_c$ are normalized on πT_c Matsubara frequencies, $\gamma_B=R_B/\rho_n\xi$ is the suppression parameter, R_B is the specific resistance of the SN interface, G_s is a normal Green function in superconducting electrode, Δ is the magnitude of the electrodes pair potentials and $+\frac{\varphi}{2}$ and $-\frac{\varphi}{2}$ are their phases.

From the definition [2] of the parameter $\gamma_B=R_B/\rho_N\xi_N=2\ell_N/3\xi_N\langle D/(1-D)\rangle$, where R_B is specific boundary resistance, ρ_N, ξ_N, ℓ_N are resistivity, decay length and electron mean free path, D is transparency of SN interface, it follows that the smaller is SN interface transparency the larger is the parameter γ_B. It was found experimentally [3–8] that the typical magnitude of parameter γ_B at fabricated *in-situ* Nb/Al, Nb/Cu metal interfaces is of the order unity. The value $\gamma_B=9.4$ we have to use to get the fitting of our experimental data is nearly one order of magnitude large than that obtained in [5–8] for Nb/Cu interfaces. This permits us to conclude that $\gamma_B=9.4$ indicates that our *ex-situ* made Nb/Cu interfaces are low-transparent .

The pair amplitude (2) was used as boundary condition to solve the Usadel equation [1]:

The value of γ_{BM} significantly impacts on the properties of distribution of Green functions Φ over Matsubara frequncies ω. At the small $\omega\ll\Delta$ (which has significant role at low temperatures), the pair amplitude is simplified to:

$$\Phi_\omega(\pm\infty)=\frac{\Delta}{(1+\gamma_{BM}\Delta)}e^{\pm i\varphi/2}, \tag{3}$$

Such expression provides natural division between large and small $\gamma_{BM}\approx 1/\Delta$ which is order of unity: at $\gamma_{BM}\ll 1/\Delta$ the effective pair amplitude of the overlapped region of nanowire is $\Phi_\omega(\pm\infty)=\Delta$, while in the opposite case it is significantly decreased $\Phi_\omega(\pm\infty)=1/\gamma_{BM}$, leading to significant decrease of the critical current of the junction.

In the case of $\omega \gg \Delta$ (which has more impact at high temperatures), the expression of pair amplitude has a form:

$$\Phi_\omega(\pm\infty) = \frac{\Delta}{(1+\gamma_{BM}\omega)} e^{\pm i\varphi/2}, \tag{4}$$

It is clear that the point between small and large γ_{BM} is shifted to $1/\omega$. However, even at the large temperatures, the low Matsubara frequencies give the most significant contribution in the transport properties, providing the transition between transparent and rough boundary at γ_{BM} in order of unity.

An expression for supercurrent I_S inside the metal layer is

$$\frac{eI_S(\varphi)}{\pi T \mathcal{A}} = -\sum_{\omega=-\infty}^{\infty} \frac{G_\omega^2}{\rho\omega^2} Im\left[\Phi_\omega \frac{\partial \Phi_\omega^*}{\partial x}\right]. \tag{5}$$

where ρ_n is the resistivity of the normal metal and $\mathcal{A}$ - area of the wire cross-section.

During the calculation of the model $I_C(T)$ dependence for JJ1 and JJ2 we use following procedure. We determine the geometric parameters from the image and have two free parameters: coherence length ξ and interface parameter γ_B. We make a grid in the plane (ξ, γ_B) and for each point calculate the least square root between the numerical calculation and data points (for the both JJ simultaneously). Then make the same procedure again with smaller step of the grid and take optimal point. For the reasonable region of parameters such procedure provide the only minimum and doesn't provide any ambiguity.

Calculation of the temperature T_C^{SNS} was done by following method: the $I_C(T)$ dependence is calculated by numerical solution of Eqs. (1) - (5). Then we determine by Newton method the temperature at which critical current of structure exceed $I_C^* = 3\mu A$. The threshold $I_C^* = 3\mu A$ was chosen, since below this value the experimental points significantly deviate from exponential dependence and rapidly go to zero.

Cooling power

In accordance with [9] in N metals the electron-phonon cooling power per unit volume, Q, in disordered electronic systems can be calculated by the following expressions.

At small temperatures

$$T < T_* = \frac{\hbar c_t}{k_B \ell} \tag{6}$$

Q is given by

$$Q = \frac{32\pi^4}{945}\frac{N_0 c_t \tau}{\rho_0 \ell^6}\left(\frac{p_F^2}{m}\right)^2\left(1+\frac{c_t^5}{c_l^5}\right)\frac{T^6}{T_*^6},$$

where ℓ is electron mean free path, N_0 is density of states at Fermy level, ρ_0 is the mass density of the material, c_l, c_t, are longitudinal and transverse speeds of sound, p_F is momentum Fermy, m is electron mass, $\tau = \ell/v_F$, $v_F = p_F/m$.

In the opposite limit

$$T \gg T_*,$$

the electron-phonon cooling power per unit volume is

$$Q = \frac{32\pi^4}{945}\frac{N_0 c_t \tau\hbar}{\rho_0 \ell^6}\left(\frac{p_F^2}{m}\right)^2\left(\frac{3\zeta(5)}{3\pi}\frac{c_t^4}{c_l^4}\frac{T^5}{T_*^5}+\frac{4\pi^2}{45}\frac{T^4}{T_*^4}\right). \tag{7}$$

For a Cu film with a thickness, $d_n \approx \ell = 10$ nm and transverse speed of sound $c_t = 2.3$ km/s and $c_t = 4.8$ km/s the characteristic temperature

$$T_* = \frac{\hbar c_t}{k_B \ell} \approx 1.8K. \tag{8}$$

For our working temperature $T \approx 4,2$ K, the ratio $T/T_* \approx 2,3$, and the first term in parentheses of expression (7) is more than two order of magnitude smaller compare to the second term.

$$\frac{3\zeta(5)}{3\pi}\frac{c_t^4}{c_l^4}\frac{T}{T_*} = \frac{1,037}{\pi}\left(\frac{2,3}{4,8}\right)^5 2,3 = 0,019$$

$$\frac{16\pi^2}{45} = 3,5$$

Therefore at $T \approx 4,2$ K for Q we may take only the last item in (7)

$$Q = \frac{32\pi^4}{945} \frac{N_0 \tau k_B T_*}{\rho_0 \ell^5} \left(\frac{p_F^2}{m} \right)^2 \frac{4\pi^2}{45} \frac{T^4}{T_*^4}. \tag{9}$$

---

[1] K. D. Usadel, *Phys. Rev. Lett.* **25**, 507 (1970).
[2] Kuprianov, M. Y. & Lukichev, V. F., *Sov. Phys. JETP* **67**, 1163 (1988).
[3] Zehnder, A., Lerch, P., S. P. Zhao, T. Nussbaumer, E. C. Kirk, & H. R. Ott, *Phys. Rev. B* **59**, 8875 (1999).
[4] Brammertz, G., Poelaert, A., Golubov, A. A., Verhoeve, P., Peacock, A. & Rogalla, H., *J. Appl. Phys.* **90**, 355 (2001).
[5] Baxter, D. V., Steenwyk, S. D., Bass, J. & Pratt, W. P., *J. Appl. Phys.* **85**, 4545 (1999).
[6] Park, W., Baxter, D. V., Steenwyk, S., Moraru, I., Pratt, W. P. & Bass, J., *Phys. Rev. B* **62**, 1178 (2000).
[7] Sauvageau, J., Ono, R., Jain, A., Li, K. & Lukens, J., *IEEE Tran. Magn.* **21**, 854 (1985).
[8] V. S. Stolyarov *et al*, *Nature Comm.* **2**, 2277 (2018).
[9] Nikolic, D., Basko, D. M. & Belzig, W., *Phys. Rev. B* **102**, 214514 (2020).
